# Changing epidemiology of dengue in Sri Lanka—Challenges for the future

**DOI:** 10.1371/journal.pntd.0009624

**Published:** 2021-08-19

**Authors:** Gathsaurie Neelika Malavige, Chandima Jeewandara, Azhar Ghouse, Gayasha Somathilake, Hasitha Tissera

**Affiliations:** 1 Centre for Dengue Research, University of Sri Jayewardenepura, Nugegoda, Sri Lanka; 2 Allergy Immunology and Cell Biology Unit (AICBU), Department of Immunology and Molecular Medicine, University of Sri Jayewardenepura, Nugegoda, Sri Lanka; 3 Epidemiology Unit, Ministry of Health, Colombo, Sri Lanka; University of Heidelberg, GERMANY

## Abstract

**Background:**

Dengue infections are on the rise in Sri Lanka and are spreading to all areas in the country. Here, we discuss the changes in dengue epidemiology in Sri Lanka in relation to changes in age distribution, changes in seroprevalence rates over time, and possible reasons contributing to such changes.

**Methods and findings:**

Although the incidence of dengue increased 20-fold from the year 2000 to 2012 and a further 3-fold from 2012 to 2019, this increase is not reflected in a similar increase in the age-stratified seropositivity rates for dengue. For instance, the annual seroconversion rates were 0.76% in 2013 and 0.91% in 2017. The annual seroconversion rates in the 6 to 17 age group were 1.5% per year in 2003, 3.9% in 2013, and 4.1% in 2017. In addition, although a 13-fold increase in dengue was seen in those who were <19 years of age, a 52.4-fold increase was seen in the 40- to 59-year age group. The case fatality rates (CFRs) have similarly changed, with 61.8% of deaths occurring in those <19 years of age in the year 2000, while in 2012 to 2018, the highest CFR were seen in those who were aged 20 to 39 years. Although there has been a marked increase in the number of cases, the vector densities did not change during a 4-year period. The proportion of adult individuals experiencing a secondary dengue infection has also remained between 65% and 75% between the years 2004 and 2018.

**Conclusions:**

A change in the ratio of symptomatic to asymptomatic infections can give rise to changes in the reported incidence of dengue. In order to take an appropriate policy decision in dengue control activities, it would be important to study the changes in virus serotypes, vector dispersion, and densities. Further, the contribution of the rise in metabolic diseases to an increase in the symptomatic as well as more severe infections due to dengue is explored.

## Author summary

Dengue infections are on the rise in Sri Lanka and are spreading throughout the country. Although the incidence of dengue increased 20-fold from the year 2000 to 2012 and a further 3-fold from 2012 to 2019, this increase is not reflected in a similar increase in the age-stratified seropositivity rates for dengue, with the annual seroconversion rates being 0.76% in 2013 and 0.91% in 2017. In addition, although a 13-fold increase in dengue was seen in those who were <19 years of age, a 52.4-fold increase was seen in the 40- to 59-year age group. Despite the rise in the number of cases, the vector densities have remained unchanged in recent years. Therefore, assessing the changes in the factors such as the ratio of symptomatic to asymptomatic infections, virus serotypes, comorbid factors, and the serostatus of individuals are important considerations.

## Introduction

Dengue viral infections are the most rapidly emerging mosquito-borne viral infection in the world, resulting in approximately 390 million infections annually, of which 96 million are inapparent [1]. WHO named dengue as one of the 10 threats to global health in 2019 due to the burden of infection in low- and middle-income countries, and, also, as there has been a 15-fold increase in dengue over the last 2 decades [2]. The annual global cost of dengue has been estimated to be a staggering US$8.9 billion in 2013 [3]. Costs of dengue control activities and hospitalization were estimated to be US$3.45 million in year 2012, in Sri Lanka [4], which highlights the economic burden due to this disease in resource-poor countries. The case fatality rates (CFRs) due to dengue have declined in Sri Lanka from approximately 1% in 2009 to <0.3% in 2018 [5]. This was achieved through early presentation and diagnosis and timely hospital admission for detection of plasma leakage and meticulous fluid management in hospitals to prevent deaths due to shock or fluid overload. However, the incidence of dengue and more severe forms of dengue continues to rise.

**Fig 1:**
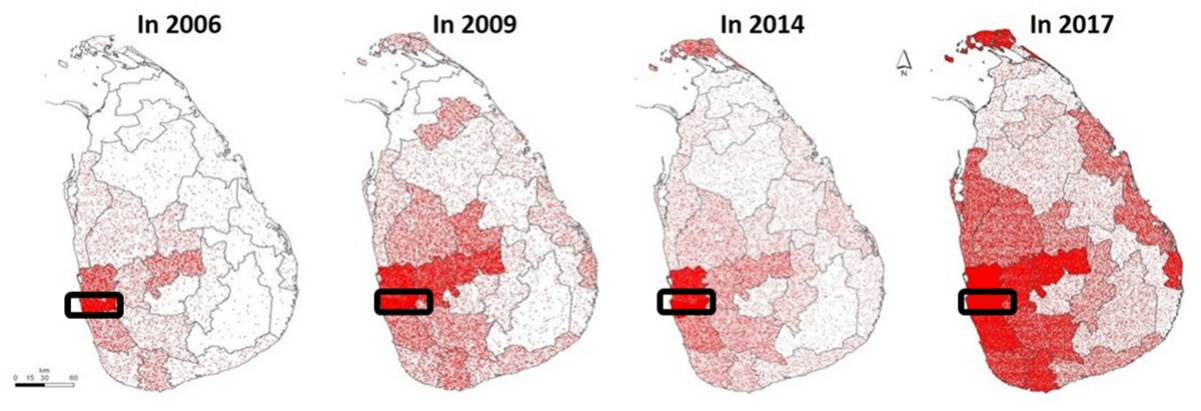
Geographic spread and increase in the number of cases of dengue infection from 2006 to 2017 in Sri Lanka. Each red dot denotes a reported case by each district. Colombo district is circled in black [15]. *Source of the data*: www.epid.gov.lk.

There is evidence that dengue viruses (DENVs) have been circulating in Sri Lanka during the 1960s, and first outbreaks of dengue infections were reported in 1965 (26 cases and 6 deaths) and in 1972 (27 cases) [6,7]. An island-wide seroepidemiological survey that was carried out from 1966 to 1967 showed that antibodies to the DENV were seen in a large proportion of individuals [8]. Therefore, it is evident that the population was exposed to dengue infections in the past without the occurrence of outbreaks of dengue hemorrhagic fever (DHF), which is a severe form of dengue. The initial large outbreak of DHF in Colombo, the capital of Sri Lanka, occurred in 1989, with 206 clinically diagnosed cases of dengue and 20 deaths (CFR 9.8%), followed by in 1990, 1,080 cases with 60 deaths (CFR 5.5%) [9]. Since then, the reported incidence of dengue has increased every 3 to 5 years, with a gradual spread to many parts of the country (Fig 1). Until 2009, the number of cases reported was less than 15,000/year and increased to over 40,000/year in 2009, which was attributed to the emergence of DENV serotype 1 [10]. DENV-2 and DENV-3 were not detected from the year 2009 to mid-2016, although the predominant circulating serotypes prior to the year 2009 were DENV-2 and DENV-3 [10–12]. The largest reported epidemic of dengue was seen in 2017, with a total number of 186,101 cases being notified with 440 deaths, which coincided with the reemergence of DENV-2 [13]. Although fewer cases were reported in 2018 (51,659 cases) compared to 2017, there was again another surge in 2019, with 105,049 cases reported [14]. Here, we discuss the changes in dengue epidemiology in Sri Lanka in relation to age distribution, seroprevalence rates over time, and possible reasons for such changes. The changes highlighted by this manuscript would be helpful in making more informed decisions regarding the control of dengue and to influence policy decisions.

Dengue has been a notifiable disease since 1996 as part of the national integrated communicable disease surveillance system. Epidemiological data capture symptomatic dengue patients seeking healthcare who were classified according to a standard case definition based on the 1997/2011 WHO classification [16]. Although more recently, patients are screened using NS1 rapid antigen or dengue antibody assays, given the limited diagnostic test availability, especially in the periphery, many cases are diagnosed clinically and with simple laboratory investigations using the surveillance case definition [11].

### Changes in the age distribution of morbidity and mortality rates of dengue infections in Sri Lanka

**Fig 2:**
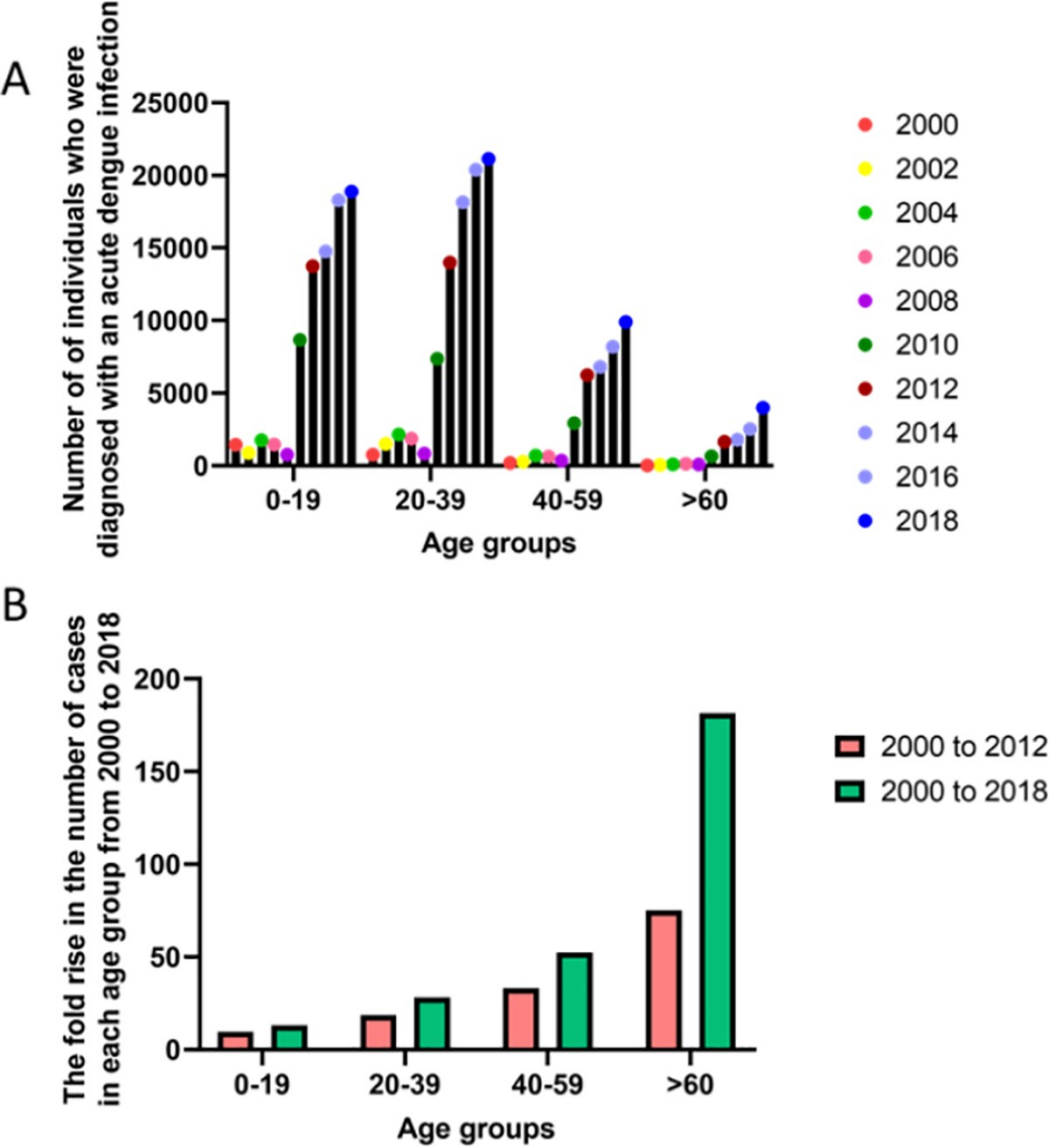
The increase in the prevalence of dengue cases from 2000 to 2018 in different age groups in Sri Lanka. The number of dengue cases reported from 2000 to 2018 in each age group **(A)** and the fold rise of the number of cases from year 2000 to 2012 and 2018 **(B)**.

When dengue epidemics initially began to occur in 1989, they were predominantly childhood infections. For instance, in the year 2000, 1,444 (59.9%) of the cases were in children <19 years of age, with 27.6% of the infections occurring in children <9 years of age. The proportion of childhood infections have declined in recent years, with 13,736 (38.7%) of the cases occurring in children <19 years of age (17.2% in <9 years old) in 2012 and 18,878 (35.7%) in <19-year-old individuals (16% in children <9 years old) in 2018 (Fig 2A). In addition, there was a disproportionate rise in the number of cases in the different age groups. In children <19 years, the number of infections from 2000 to the year 2012 has increase 9.5-fold and while only a 1.4-fold increase was seen from 2012 to 2018. In contrast, the increase in the 20 to 39 age group is 18.6-fold from 2000 to 2012 and 1.5-fold from 2012 to 2018 (Fig 2B). In the >60-year age group, there was a 75-fold rise from 2000 to 2012 and a 2.4-fold rise from 2012 to 2018. Therefore, based on this data, although the incidence of dengue infections has increased over the years in all age groups, the steepest rise has been in the older age groups. Although the reasons for the rise in dengue cases in the adult population are not clear, it could be due to multiple factors such as the differences between infection with different DENV serotypes, change in host factors (increase in comorbid illnesses), and the intensity of transmission of the DENV.

**Fig 3:**
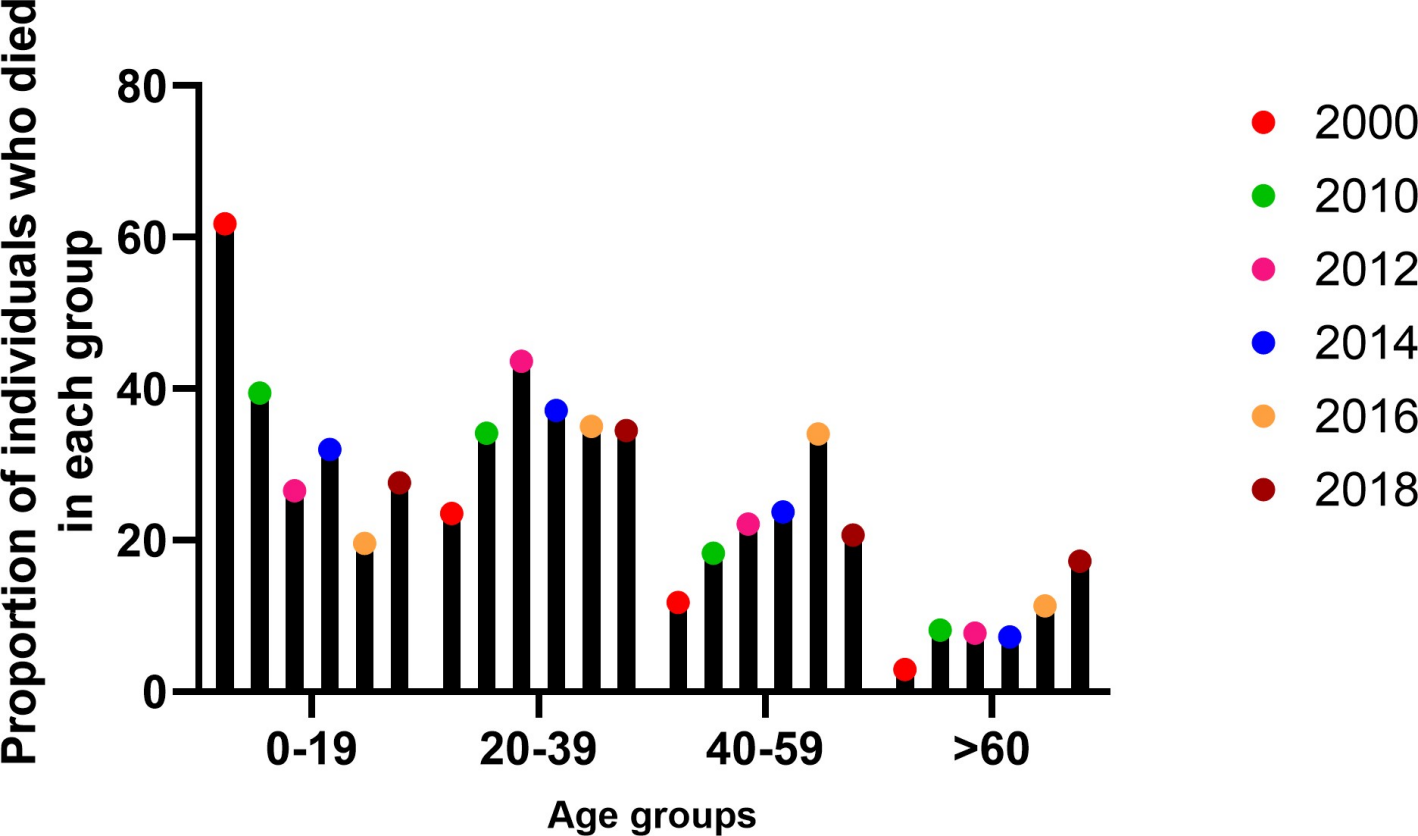
The change in the CFRs in different age groups from the year 2000 to 2018. CFR, case fatality rate.

Changes in the age distribution of patients with dengue have also been reflected in the reported deaths among the different age groups. In 2000, the highest CFRs were seen among children, with 61.8% of deaths occurring in those <19 years of age. Among these, the highest CFR was seen in the 1- to 4-year age group with 32.35% of deaths, followed by 17.6% in the 5 to 9 age group. Currently, the highest CFR is seen in the young adult group (20 to 39 years), accounting for 35.05% of all CFRs in 2012 and 34.48% in 2018 (Fig 3). This trend was seen even during the largest outbreak experienced in Sri Lanka in 2017, with 34.8% of all deaths occurring in the 20 to 39 age group. During recent years, the proportion of deaths in the older age group are also increasing, with 17.5% of all deaths in 2017 occurring in the over 60 age group and 17.2% in 2018. The increase in deaths in the older age groups could be a result of increase in infection of older individuals who have multiple comorbidities, which are associated with the development of more severe forms of dengue [17,18]. Therefore, the occurrence of deaths among the older age group could be reduced by public education programs of the risk factors of severe dengue and those with risk factors to seek early medical care for timely interventions.

### Changes in age-stratified seroprevalence rates

**Fig 4:**
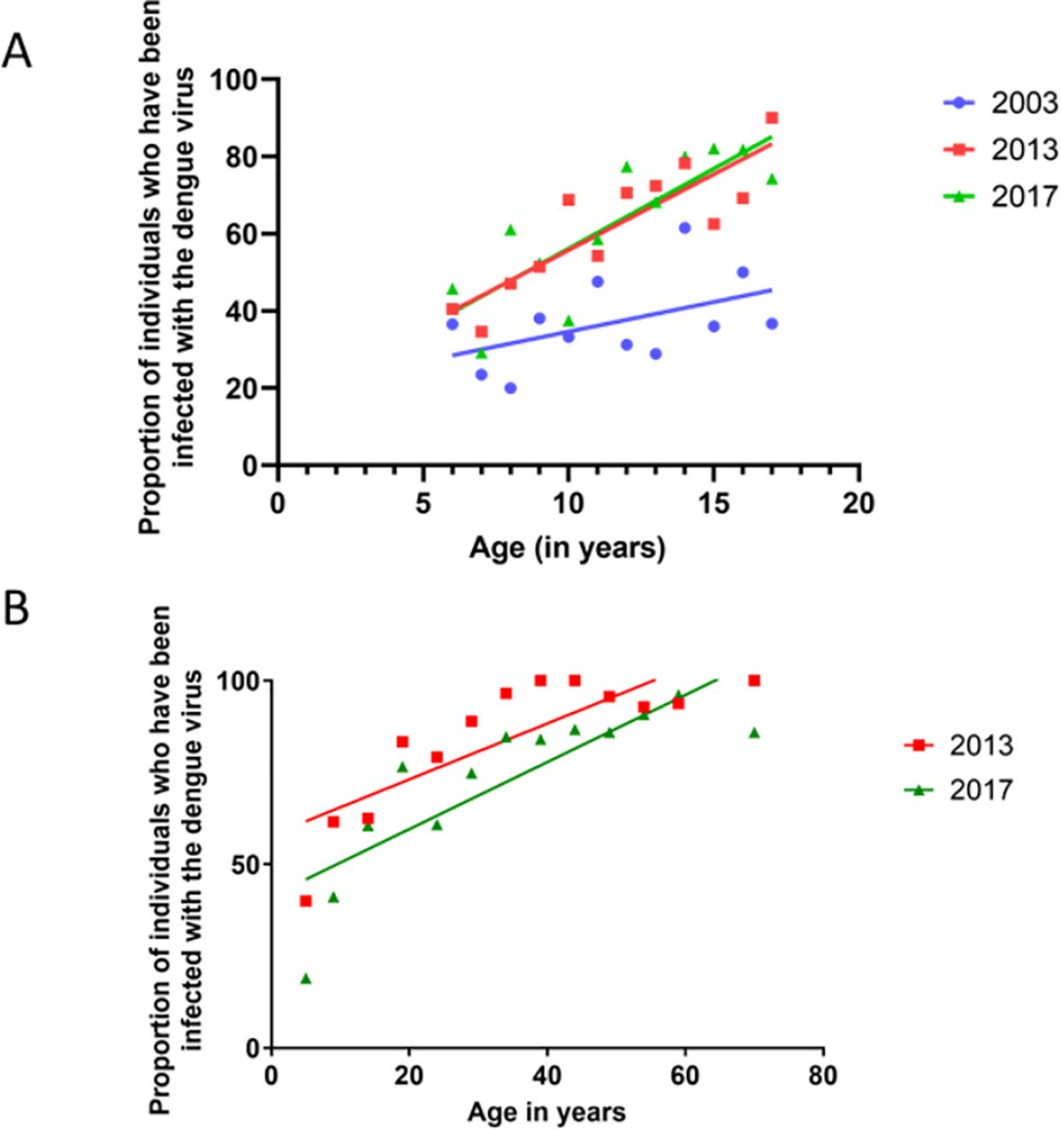
Age-stratified seroprevalence in the Colombo district in 2003, 2013, and 2017. The age-stratified seroprevalence was determined in children aged 6 to 17 years in 2003, 2013, and 2017 **(A)** and in individuals aged 6 to 80 in 2013 and 2017 **(B)**.

Although dengue infections are currently reported from all districts in the country, initial epidemics in the 1990s and early 2000 were confined mostly to the Western Province and the Colombo district. The Western Province is the most populous in Sri Lanka, compromising of 3 major districts, which specifically includes the Colombo district, the national capital, reporting the highest dengue burden. In 2000, 49.7% of cases were reported from the Colombo district, and it was 22.5% in 2012 and 19.8% in 2019 [19]. As the incidence of dengue has been the highest in the Colombo district since initial dengue epidemics and due to the steady rise in the number of cases annually, it would be reasonable to presume that the increase in the number of cases over the years is likely to be due to more intense transmission. This would be reflected in an increase in the proportion of dengue seropositive individuals in different age categories over the years. We conducted an age-stratified seroprevalence study among children in 2003 [20], another study in all age groups in 2013 [21], and again in 2017 (the methods for the serosurveillance in 2017 is shown in S1 Methods as a protocol). As shown in Fig 4A, the age-stratified seroprevalence had increased from 2003 to 2013 (10 years), but there was no major difference from 2013 to 2017 (5 years). The annual seroconversion rates in the 6 to 17 age group in 2003 was 1.5% per year, whereas it increased to 3.9% by 2013 and 4.1% by 2017 (Fig 4A).

We also compared the seroprevalence rates in 2013 versus 2017 (5-year gap), and the annual seroconversion rates in the overall population were 0.76% per annum in 2013 and 0.91% per annum in 2017 (Fig 4B). Therefore, despite the marked rise in the number of dengue cases since mid-2016, the age-stratified seroprevalence rates in the whole population have not changed over 5 years. Overall, the data appear to suggest that a higher transmission rate, reflected by an increase in the age-stratified seroprevalence alone, does not appear to account for the continuous rise in the number of cases. It was recently shown that vector densities in many districts in Sri Lanka between the 2013 and 2017 time period was similar throughout this time period [13]. Therefore, more recent dispersion of vectors and higher vector densities are unlikely to have led to the exponential increase in the number of cases in Sri Lanka.

### Rise in the incidence of dengue in Sri Lanka

**Fig 5:**
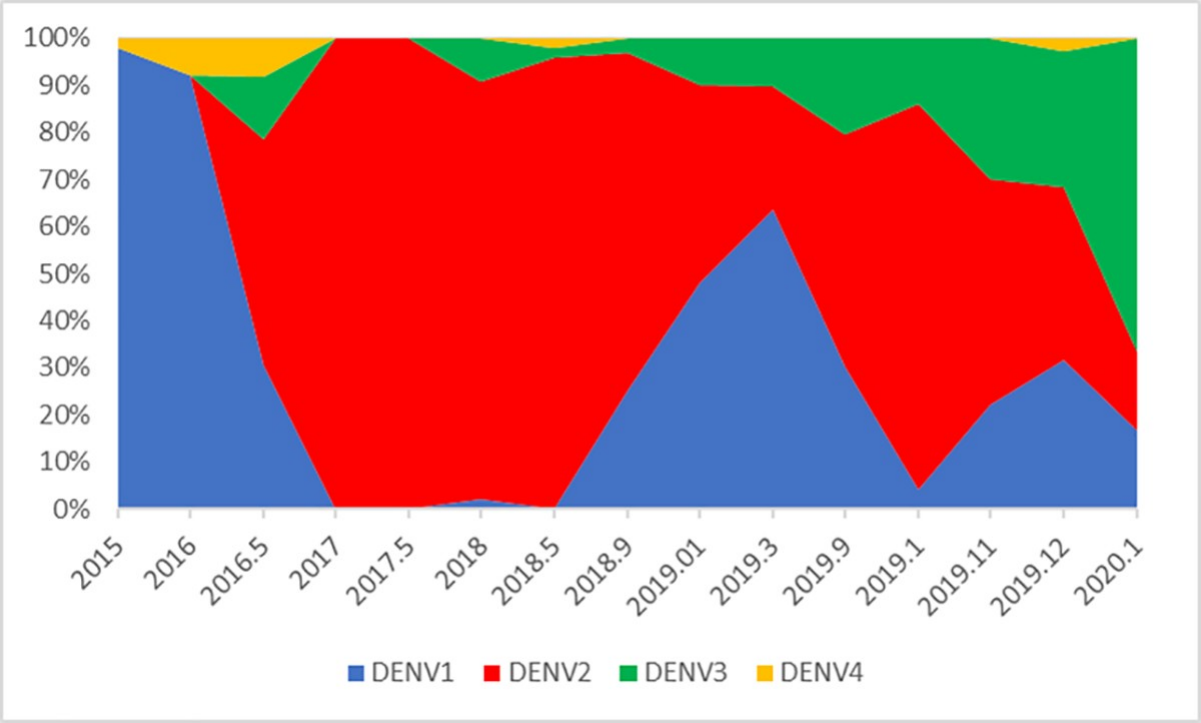
Changes in the circulating DENV serotypes in Colombo, Sri Lanka from 2015 to January 2020. DENV, dengue virus.

Although a rise in the incidence of dengue is seen in almost all dengue-affected countries in the world, it is now predominantly affecting adults in many Western Pacific countries and causing higher mortality in adults, as described earlier. Since the rise in the incidence of dengue does not appear to be due to higher transmission of dengue alone, there could be several other factors that contribute to this increase. The incidence of dengue reflects the number of symptomatic or apparent dengue infections. The ratio of asymptomatic to symptomatic cases has shown to vary from 2.1:1 to 13:1 [22–24]. Previous studies from the Colombo district Sri Lanka have shown that the asymptomatic to symptomatic ratio was 3.4:8.4 between 2008 and 2010 [25]. Therefore, in addition to the increase in transmission of dengue, the change in the proportion of symptomatic to asymptomatic infections could be one of the main contributors to the rise in the incidence of dengue. Many factors could affect the ratio of symptomatic to asymptomatic infections including changes in the predominant circulating virus serotype. The sudden increase in dengue infections in 2009 coincided with the emergence of a new DENV-1 genotype in Sri Lanka [26]. In addition, the unprecedented epidemic seen in Sri Lanka during 2017 and the increase in the number of cases seen toward the latter months of 2019 coincides with the introduction of DENV-2 and DENV-3, respectively (Fig 5).

In the majority, initial infection with one of the 4 DENV serotypes results in asymptomatic or mild undifferentiated fever, whereas secondary dengue infections (subsequent infection with a heterologous DENV serotype) have shown to increase dengue disease severity [27–29]. Symptomatic dengue is a dengue infection in which individuals develop clinical features suggestive of an acute dengue infection (fever, myalgia, and arthralgia), which may or may not be supported by a confirmatory test in a dengue-endemic country. Those who have asymptomatic dengue (or as some studies have named it as inapparent dengue) are those who do not have any symptoms when infected with dengue or have nonspecific symptoms [1]. A large community study carried out in Nicaragua has shown that inapparent dengue infection occurs in an equal proportion of those experiencing a primary or secondary dengue infection [30]. In Sri Lanka, the proportion of adults who are experiencing a secondary dengue infection, admitted to tertiary care hospitals in Colombo, has more or less remained the same over the years. For instance, in 2004, 65.5% of adults with an acute dengue infection, admitted to tertiary care hospitals in Colombo, were experiencing a secondary dengue infection [31]. The proportion of secondary dengue infection increased to 80.4% by 2010 [32], but then declined to 75% by 2011 [10], 70% in 2015 [33], and 64.9% in 2018 [34]. However, serostatus and severity of infection of those admitted to hospitals would not necessarily reflect the serostatus of those who acquire the infections in the community. Furthermore, the clinical manifestations of dengue infection could be different in children compared to adults. For instance, studies have shown that secondary dengue infection is more likely to result in DHF in children than in adults [28,35,36]. However, primary infection is more likely to be symptomatic in adults compared to children [13]. It is possible that the increase in the incidence of dengue is due to a higher number of individuals experiencing a secondary dengue infection, as such infections are more likely to be symptomatic than primary dengue. Such information can only be derived from carefully conducted, longitudinal studies in the community. However, as the proportion of secondary dengue infections in adults being admitted to hospitals has remained unchanged over the years, there could be many other contributing factors that lead to a continuous upward trend in the cases of dengue infection among adults.

Metabolic diseases such as the presence of diabetes, hypertension, and obesity are known to be independent risk factors that are associated with DHF [37–39], and diabetes has also shown to be an independent risk factor for shock and severe organ involvement in acute dengue [17,18]. Therefore, as the presence of diabetes and such metabolic diseases increase dengue disease severity in symptomatic patients, they could also increase the likelihood of occurrence of symptomatic infection, rather than asymptomatic infection, when infected with the DENV. However, the effect of diabetes and metabolic disease in the likelihood of increasing the probability of developing a symptomatic infection has not been studied. Careful studies are needed to determine if the presence of diabetes or metabolic disease at the time of infection with the DENV increases the likelihood of developing a symptomatic disease.

There has been a marked rise in the prevalence of diabetes and other metabolic diseases in Sri Lanka during the past 30 years. For instance, the prevalence of diabetes in the Western Province of Sri Lanka, which was 5.02% in 1990, increased to 6.5% in 2000, 16.4% in 2006 [40], and further rose to 27.6% by 2015 [41]. When conducting the seroprevalence studies in 2003 and 2013, data regarding the height and weight of individuals were also recorded, and the body mass index (BMI) was calculated. In children, BMI was plotted on the Centers for Disease Control and Prevention (CDC) BMI for age growth charts (for boys or girls) to acquire the percentile ranking as it was the most suitable indicator for describing growth patterns in children [42]. Accordingly, children with a BMI centile of ≥95% were classified as being obese. In 2003, 37/575 (6.43%) of the children studied were classified as obese, and this increased to 59/599 (9.85%) by 2013, which was a significant increase (*p* = 0.03). Therefore, during a period of 10 years, the proportion of obese children has significantly increased, and the prevalence of diabetes among adults had increased from 6.5% in 2000 to 27.6% by 2015 [41]. Such marked increases in obesity and diabetes in the community may have potentially affected the ratio of symptomatic and asymptomatic infections, and, thereby, contributing to the increase in the number of cases. The increase in the number of cases leading higher CFR in the older age groups is likely to be due to the occurrence of more severe disease in those with comorbidities.

In summary, despite extensive vector control measures, dengue infections are on the rise in Sri Lanka and are spreading to all geographical locations in the country. There is a disproportionate rise in the cases, and deaths are more frequently seen in the older age groups. Furthermore, despite the incidence of dengue rising 20-fold from year 2000 to 2012 and a further 3-fold from 2012 to 2019, this increase is not reflected in a similar pattern in the age-stratified seropositive rates. As a change in the ratio of symptomatic to asymptomatic infections can also give rise to changes in the incidence of dengue, it would be important to carefully study all contributing factors such as changes in virus serotypes, vector densities, health-seeking behaviors of adults versus children, and whether the rise in metabolic diseases could also contribute to such changes. This can be further ascertained by carefully conducted and innovative longitudinal community studies.

## Supporting information

S1 Methods Supporting information methods. (DOCX)
