# Supplementary material for: Changing epidemiology of dengue in Sri Lanka—Challenges for the future
Source: PLoS Negl Trop Dis. 2021 Aug 19;15(8):e0009624. doi: 10.1371/journal.pntd.0009624 (PMC8375976; doi:10.1371/journal.pntd.0009624)
Supplement: S1 Methods — (DOCX) [file pntd.0009624.s001.docx]

**Supplementary methods**

This study was designed to assess the seroprevalence of dengue infection in the Colombo district, Sri Lanka. The study was carried out during a six-month period from March to August 2017. It was a community-based cross-sectional descriptive study of 1,625 participants using an age stratified (1 to 69 years of age), multistage cluster sampling method. The total population in the Colombo district was stratified into metropolitan, urban and rural sectors of residence. Sixty-four clusters (64) from a total of 557 smallest administrative areas to be selected from each sector of residence were determined according to probability proportional to the size of the population in each sector. After obtaining written informed consent, three milliliters of venous blood was collected from each study participant into clearly labeled collection tubes subsequent to the completion of an interviewer-administered questionnaire. A qualitative assay of Dengue IgG serology was carried out using the indirect dengue IgG capture ELISA (PanBio), at the Centre for Dengue Research at the University of Sri Jayewardenepura, Sri Lanka. Dengue IgG ELISA testing was performed as per the manufacturer’s instructions.

The ethical clearance for this study was obtained from the Ethical Review Committee of the Faculty of Medicine, University of Colombo.

**Table A: Dengue Classification used in Sri Lanka,1997 - 2020**

| Dengue Classification | 1997 | 2011 |
| --- | --- | --- |
| Undifferentiated fever | Fever with maculopapular rash | Fever with maculopapular rash +/- Resp and Gastro-Intestinal symptoms |
| Dengue Fever | Fever with two or more nonspecific features – headache especially retro-orbital pain, arthralgia/ myalgia, rash (defuse, erythematous, macular), and hemorrhagic manifestations  + Leucopenia | Fever with two or more nonspecific features, haemorrhagic manifestations  + leucopenia (≤5000 cells/µl) and Thrombocytopenia (<150x10^9^/L) |
| Dengue Haemorrhagic Fever (Without Shock) | Acute fever history, hemorrhagic tendencies, Thrombocytopenia (<100x10^9^/L), Evidence of plasma leakage (HCT ≥20%), pleural effusion, ascites, and hypoproteinaemia | Acute fever history, hemorrhagic manifestations, Thrombocytopenia (<100x10^9^/L), Objective evidence of plasma leakage due to increased vascular permeability – shown by rising HCT / haemoconcentration (HCT ≥20%) or evidence of pleural effusion, ascites, or hypoproteinaemia/albuminaemia |
| Dengue Hemorrhagic Fever (with Shock) | All four above, + evidence of circulatory failure ( PP ≤20mmHg, Hypotension) | Evidence of Plasma leakage + circulatory failure (weak pulse, PP ≤20mmHg), hypotension OR profound shock with undetectable BP |
| Expanded Dengue Syndrome | Organopathy + | Organopathy + |
| Laboratory tests offered (selectively for subset of cases) | HAI, IgM ELISA, RT-PCR | RDT NS1, RDT IgM, IgM/IgG ELISA, RT-PCR |
| Reference | WHO Geneva, 1997 (1) | WHO SEARO 2011 (2) |
